# BNT162b2 mRNA COVID-19 Vaccine Does Not Impact the Honeymoon Phase in Type 1 Diabetes: A Case Report

**DOI:** 10.3390/vaccines10071096

**Published:** 2022-07-08

**Authors:** Marco Infante, Andrea Fabbri, Nathalia Padilla, Francesca Pacifici, Pasquale Di Perna, Laura Vitiello, Alessandra Feraco, Maria Giuliano, Marina Passeri, Massimiliano Caprio, Camillo Ricordi, David Della-Morte, Luigi Uccioli

**Affiliations:** 1 CTO Andrea Alesini Hospital, Division of Endocrinology and Diabetes, Department of Systems Medicine, University of Rome Tor Vergata, Via San Nemesio 21, 00145 Rome, Italy; pasquale.diperna@aslroma2.it (P.D.P.); maria.giuliano@aslroma2.it (M.G.); marina.passeri@aslroma2.it (M.P.); luccioli@yahoo.com (L.U.); 2 Cell Transplant Center, Diabetes Research Institute (DRI), University of Miami Miller School of Medicine, 1450 NW 10th Ave, Miami, FL 33136, USA; cricordi@med.miami.edu; 3 Section of Diabetology, UniCamillus, Saint Camillus International University of Health Sciences, Via di Sant’Alessandro 8, 00131 Rome, Italy; 4 Network of Immunity in Infection, Malignancy and Autoimmunity (NIIMA), Universal Scientific Education and Research Network (USERN), Via Cola di Rienzo 28, 00192 Rome, Italy; 5 Department of Systems Medicine, University of Rome Tor Vergata, Via Montpellier 1, 00133 Rome, Italy; andrea.fabbri@uniroma2.it (A.F.); pacifici.francesca@gmail.com (F.P.); david.dellamorte@uniroma2.it (D.D.-M.); 6 Network of Immunity in Infection, Malignancy and Autoimmunity (NIIMA), Universal Scientific Education and Research Network (USERN), Colonia Centroamérica L-823, Managua 14048, Nicaragua; nathalia.padilla22@gmail.com; 7 Laboratory of Flow Cytometry, IRCCS San Raffaele, Via di Val Cannuta 247, 00166 Rome, Italy; laura.vitiello@sanraffaele.it; 8 Laboratory of Cardiovascular Endocrinology, IRCCS San Raffaele, Via di Val Cannuta 247, 00166 Rome, Italy; alessandra.feraco@sanraffaele.it (A.F.); massimiliano.caprio@sanraffaele.it (M.C.); 9 Department of Human Sciences and Promotion of the Quality of Life, San Raffaele Roma Open University, Via di Val Cannuta 247, 00166 Rome, Italy; 10 Department of Neurology, Evelyn F. McKnight Brain Institute, University of Miami Miller School of Medicine, 1120 NW 14th St, Miami, FL 33136, USA

**Keywords:** T1D, honeymoon phase, clinical remission, autoimmunity, hyperglycemia, beta cell, C-peptide, COVID-19 vaccine, SARS-CoV-2 BNT162b2 mRNA vaccine, COVID-19 vaccination-induced hyperglycemia

## Abstract

Type 1 diabetes (T1D), which is caused by the autoimmune destruction of insulin-secreting pancreatic beta cells, represents a high-risk category requiring COVID-19 vaccine prioritization. Although COVID-19 vaccination can lead to transient hyperglycemia (vaccination-induced hyperglycemia; ViHG), its influence on the course of the clinical remission phase of T1D (a.k.a. “honeymoon phase”) is currently unknown. Recently, there has been an increasing concern that COVID-19 vaccination may trigger autoimmune phenomena. We describe the case of a 24-year-old young Italian man with T1D who received two doses of the BNT162b2 mRNA (Pfizer-BioNTech) COVID-19 vaccine during a prolonged honeymoon phase. He experienced a transient impairment in glucose control (as evidenced by continuous glucose monitoring) that was not associated with substantial changes in stimulated C-peptide levels and islet autoantibody titers. Nonetheless, large prospective studies are needed to confirm the safety and the immunometabolic impact of the BNT162b2 vaccine in T1D patients during the honeymoon phase. Thus far, T1D patients who are going to receive COVID-19 vaccination should be warned about the possible occurrence of transient ViHG and should undergo strict postvaccination surveillance.

## 1. Introduction

Since the beginning of the coronavirus disease 2019 (COVID-19) pandemic caused by the severe acute respiratory syndrome coronavirus 2 (SARS-CoV-2), it has become clear that diabetes mellitus represents a major risk factor for COVID-19 morbidity and mortality [1,2,3]. Therefore, either form of diabetes has been fairly proposed as a high-risk category requiring COVID-19 vaccine prioritization [4]. This concept also applies to type 1 diabetes (T1D), which is a chronic autoimmune disease requiring lifelong exogenous insulin therapy as a consequence of the immune-mediated destruction of insulin-secreting pancreatic beta cells [2]. Indeed, T1D adults infected with SARS-CoV-2 are at increased COVID-19-related risk [5].

The currently available COVID-19 vaccines have undoubtedly played a major role in reducing COVID-19 incidence, morbidity, and mortality [6]. However, there have been several reports of vaccination-induced hyperglycemia (ViHG) and associated complications such as diabetic ketoacidosis (DKA) and hyperosmolar hyperglycemic state (HHS) in both nondiabetic and diabetic subjects, irrespective of the vaccine type and dose number [7,8]. These patients usually presented with typical symptoms of hyperglycemia (polyuria, nocturia, and polydipsia), with some subjects reporting significant weight loss, disorientation, and lightheadedness [7].

With specific regard to T1D, different studies have shown that some patients with T1D experience a temporary instability of blood glucose levels after COVID-19 vaccine administration, which generally settles within a few days [9,10]. Nevertheless, the influence of COVID-19 vaccines on the course of the clinical remission phase of T1D (the so-called “honeymoon phase”) is currently unknown. Approximately two-thirds of patients with T1D enter the honeymoon phase shortly after the onset of clinical diabetes and insulin therapy initiation. This phase is usually accompanied by a transient, marked reduction in exogenous insulin requirements and near-normal blood glucose values (partial clinical remission) [11,12,13]. A minority of young T1D patients (about 2–12%) can also experience complete clinical remission, which is characterized by near-normal blood glucose levels without the need for insulin therapy [14]. The overall duration of the remission phase ranges widely between 1 month and 13 years [15], with an average of 7 months [16]. It has been shown that the occurrence of remission in T1D is associated with remarkable clinical advantages in the long-term, as remitters exhibit better glycemic control and lower insulin requirements during the first 5 years of follow-up [17], along with a reduced risk of chronic microvascular complications of diabetes compared to non-remitters [18]. This is probably due to the higher retention of endogenous insulin and C-peptide secretion in T1D patients who experience clinical remission [19].

The pathophysiology of the honeymoon phase is still not completely understood. Both metabolic and immune factors have been suggested to contribute to the transient beta-cell recovery associated with the honeymoon phase. Metabolic factors include an improvement in insulin sensitivity and reduced glucotoxicity due to the amelioration of glucose control attained after the initiation of insulin therapy; on the other hand, immune factors include a temporary recovery of immune tolerance to beta-cell autoantigens, which could involve T-cell regulatory pathways [11,20]. The most reliable marker of clinical remission in T1D is represented by the so-called insulin dose-adjusted HbA1c (IDAA1c), which is calculated through the following formula: HbA1c (%) + [4× insulin dose (units per kilogram per 24 h)]. An IDAA1c value of ≤9 corresponds to a predicted stimulated C-peptide value of >300 pmol/L (>0.9 ng/mL) and is considered indicative of partial clinical remission [21]. 

In this manuscript, we present the case of a patient with T1D who received the Pfizer-BioNTech COVID-19 vaccine during the honeymoon phase and subsequently experienced transient ViHG.

## 2. Case Presentation

Herein, we present the case of a 24-year-old young Italian man with T1D who received the BNT162b2 mRNA (Pfizer-BioNTech) COVID-19 vaccine during the honeymoon phase of the disease. The patient was routinely monitored at our institution (CTO Andrea Alesini Hospital, Division of Endocrinology and Diabetes, Department of Systems Medicine, University of Rome Tor Vergata). He was diagnosed with T1D in July 2020 (at 22 years of age) after the occurrence of severe hyperglycemia (501 mg/dL) and moderate DKA requiring hospitalization and treatment with intravenous insulin and fluid therapy, as per the standard of care treatment [22]. At the time of T1D diagnosis, the patient was 175 cm tall, and his body weight was 69 Kg, with a body mass index (BMI) value of 22.5 Kg/m^2^. Glycated hemoglobin (HbA1c) and fasting C-peptide values were 13.2% (121 mmol/mol) and 0.22 ng/mL, respectively. Multiple islet autoantibody positivity confirmed the diagnosis of T1D: glutamic acid decarboxylase autoantibodies (GADA), islet tyrosine phosphatase 2 autoantibodies (IA2A), and zinc transporter 8 autoantibodies (ZnT8A) were present (Supplementary Table S1). The patient denied any trauma, illness, alcohol consumption, smoking habit, medication, or illicit drug use. He also denied a family history of T1D, but he reported a family history of type 2 diabetes (maternal grandfather and paternal aunt). His past medical history was unremarkable, except for left inguinal hernia repair at 3 years of age and previous infectious mononucleosis at 15 years of age. The nasopharyngeal reverse-transcription polymerase chain reaction (RT-PCR) test for SARS-CoV-2 performed at the time of hospital admission was negative.

After DKA resolution (which occurred 48 h after the hospital admission), the patient was shortly transitioned from intravenous to subcutaneous insulin as follows: multiple daily injection (MDI) insulin therapy consisting of insulin lispro (8 IU before breakfast, 10 IU before lunch, and 8 IU before dinner) and insulin degludec [24 IU at bedtime (amounting to a total daily insulin dose of 0.7 IU/Kg body weight/24 h)]. The patient also received a consultation by a nutrition specialist, who prescribed a Mediterranean diet plan of approximately 1800 calories per day.

The patient was discharged from the hospital 5 days after admission. At the outpatient follow-up visits, the total daily insulin dose was progressively reduced due to the frequent occurrence of fasting and postprandial hypoglycemic episodes. We also prescribed the use of a subcutaneous glucose sensor (FreeStyle Libre^®^, Abbott, Chicago, IL, USA). Thirty days later, rapid-acting insulin lispro was withdrawn and low-dose basal insulin (0.09 IU/Kg body weight/24 h) was maintained (Supplementary Table S1).

Two months after DKA onset and resolution of marked hyperglycemia, the patient underwent a 120 min mixed meal tolerance test (MMTT). The fasting plasma C-peptide value was 0.74 ng/mL, whereas the peak plasma C-peptide value was 2.95 ng/mL (reached at 90 min). Supplementary Table S1 shows plasma C-peptide and glucose values, as well as C-peptide index values during the first MMTT. The C-peptide index (also referred to as the C-peptide to glucose ratio) was assessed in fasting and (MMTT)-postprandial states and was calculated through the following formula: C-peptide (ng/mL)/glucose (mg/dL) (×100) [23]. At that time, the patient had already entered the honeymoon phase, as evidenced by an IDAA1c value of 5.66. Complete blood count (CBC) and other laboratory test results (including lipid profile and markers of kidney, liver, and thyroid function, as well as markers of thyroid autoimmunity and celiac disease) were unremarkable, except for a mild vitamin D insufficiency (serum 25-hydroxyvitamin D level: 28 ng/mL) (Supplementary Table S2). We therefore prescribed calcifediol (Neodidro) at a dose of 0.266 mg every 20 days in order to correct vitamin D insufficiency and to leverage the immunomodulatory and anti-inflammatory actions of vitamin D [24,25,26,27,28]. This may be useful to halt beta-cell autoimmunity [29,30,31] and to potentially prevent or mitigate SARS-CoV-2 infection [32,33,34,35].

After 12 months (14 months after T1D diagnosis), the patient underwent a follow-up MMTT that still showed a preserved residual beta-cell function (peak plasma C-peptide value of 1.78 ng/mL reached at 120 min) (Supplementary Table S1). At that time, the patient was still on a low daily insulin dose (insulin degludec, 7 IU/day at bedtime, amounting to a total daily insulin dose of 0.10 IU/Kg body weight/24 h). Other laboratory tests performed at 12 months are shown in Supplementary Table S2.

Given the good general health status and the presence of the honeymoon phase, which only required low basal insulin daily doses, the patient was initially skeptical about the need to receive the COVID-19 vaccine, despite our medical recommendations to undergo the vaccination. He finally decided to be vaccinated against COVID-19 in December 2021. The patient received the BNT162b2 mRNA COVID-19 vaccine. The first vaccine dose was administered on 13 December 2021, whereas the second vaccine dose was administered on 3 January 2022. The patient was closely monitored after the vaccination via telemedicine (remote diabetes management using continuous glucose monitoring) and outpatient follow-up visits.

**Figure 1:**
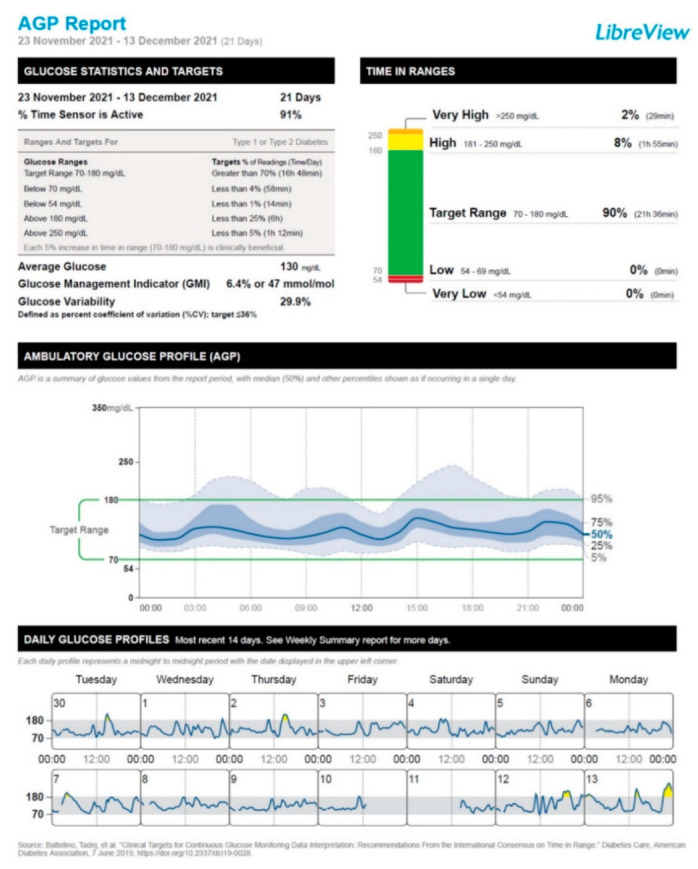
Ambulatory glucose profile (AGP) obtained from the subcutaneous glucose sensor referring to the 21 days before the first vaccine dose injection (23 November 2021 through 13 December 2021).

**Figure 2:**
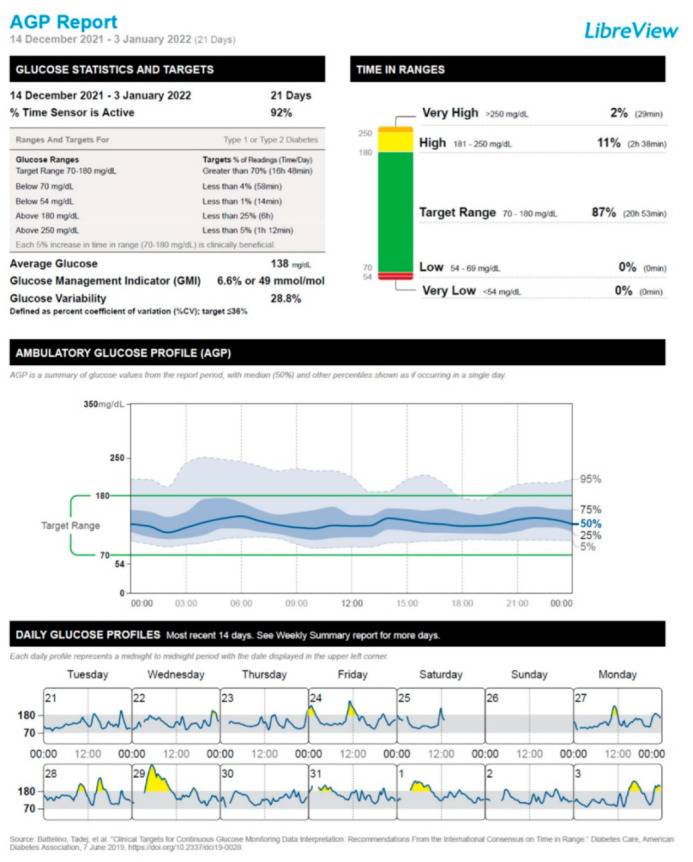
Ambulatory glucose profile (AGP) obtained from the subcutaneous glucose sensor referring to the 21 days after the first vaccine dose injection (14 December 2021 through 3 January 2022).

**Figure 3:**
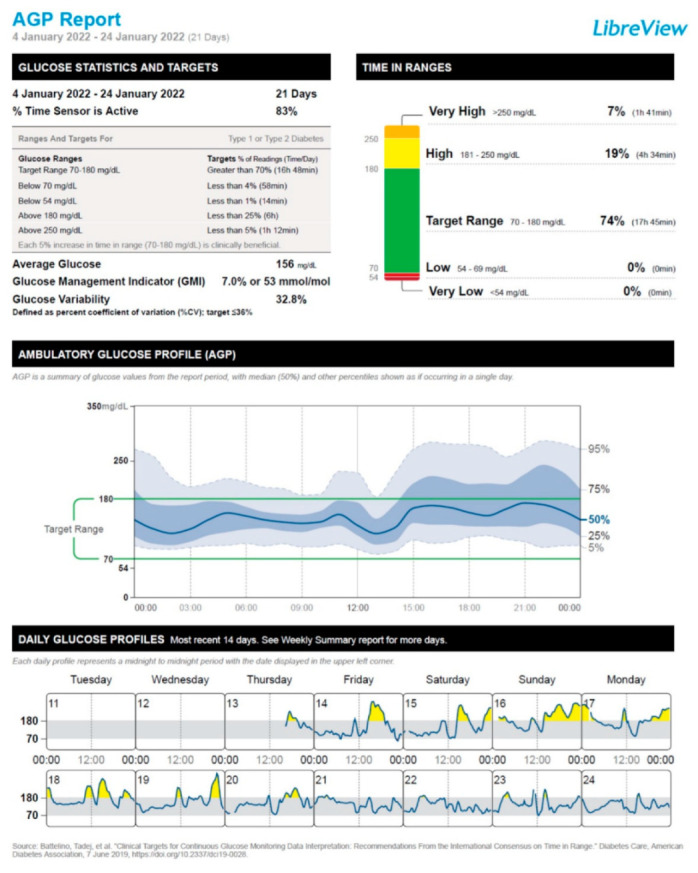
Ambulatory glucose profile (AGP) obtained from the subcutaneous glucose sensor referring to the 21 days after the second vaccine dose injection (4 January 2022 through 24 January 2022).

**Figure 4:**
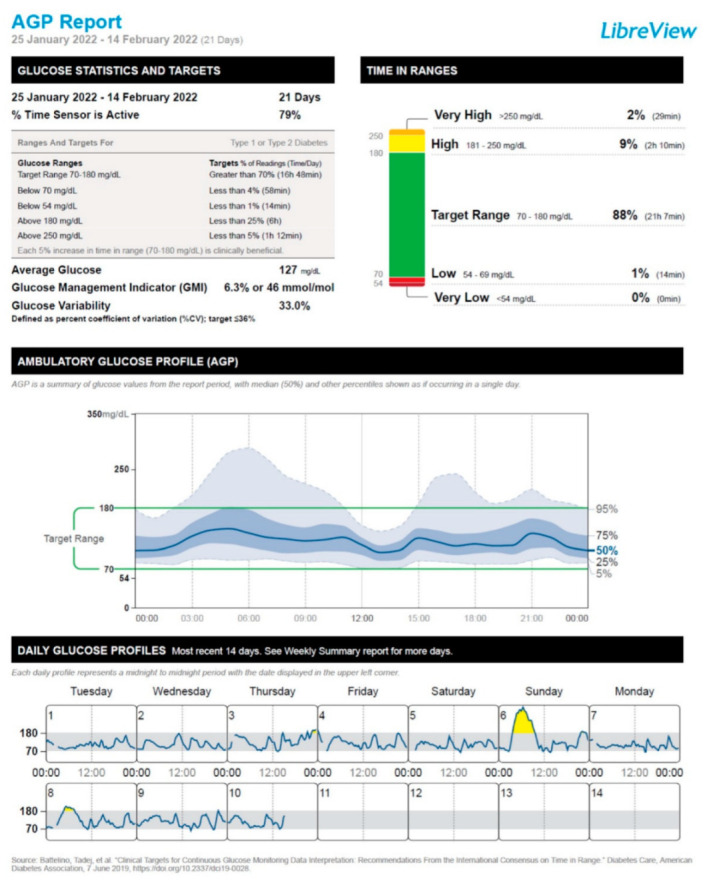
Ambulatory glucose profile (AGP) obtained from the subcutaneous glucose sensor referring to the 21-day period: 25 January 2022 through 14 February 2022.

After the administration of the first vaccine dose, the patient started to experience a deterioration in his glucose control, which mainly consisted of a moderate increase in the frequency of postprandial hyperglycemic episodes. This trend was particularly exacerbated after the administration of the second vaccine dose and resulted in the need to resume the mealtime rapid-acting insulin lispro injections for 7 days (January 14 through January 20). After the disappearance of postprandial glucose excursions, we recommended interrupting the use of rapid-acting insulin lispro. Hence, the patient remained on insulin degludec only (insulin degludec, 7 IU/day at bedtime, amounting to a total daily insulin dose of 0.10 IU/Kg body weight/24 h). Figure 1, Figure 2, Figure 3 and Figure 4 show the ambulatory glucose profile (AGP) obtained from the subcutaneous glucose sensor with regard to the following periods: (a) 21 days before the first vaccine dose injection (Figure 1); (b) 21 days after the first vaccine dose injection (Figure 2); (c) 21 days after the second vaccine dose injection (Figure 3); (d) 21 days following the previous period (c) (Figure 4). From AGP, it is noted that a deterioration of glucose control ensued from the administration of the COVID-19 vaccine, as evidenced by an increase in the time above range (TAR) 181–250 mg/dL and TAR > 250 mg/dL, by a reduction in time in range (TIR) 70–180 mg/dL, and by an increase in average glucose and glucose management indicator (GMI) values (Figure 2 and Figure 3). The worsening pattern of glucose control was particularly evident after the second vaccine dose injection (Figure 3). In particular, TIR 70–180 mg/dL decreased from 90% (before vaccination) to 74% after the second vaccine dose injection. Concomitantly, TAR 181–250 mg/dL increased from 8% (before vaccination) to 19% after the second vaccine dose injection (Figure 3; Supplementary Table S3). Subsequently, during the first 3 weeks after the second vaccine dose injection, TIR 70–180 mg/dL, TAR 181–250 mg/dL, and TAR > 250 mg/dL returned to values comparable to those observed before vaccination (Figure 4; Supplementary Table S3). Moreover, significant hypoglycemic episodes were not observed, and fasting capillary blood ketones (beta-hydroxybutyrate measured through the ketone meter Wellion Galileo^®^) remained negative (<0.6 mmol/L) at all the follow-up visits (Supplementary Table S1). The patient did not report any common or uncommon side effects or adverse events after receiving the first and second COVID-19 vaccine dose injections.

**Figure 5:**
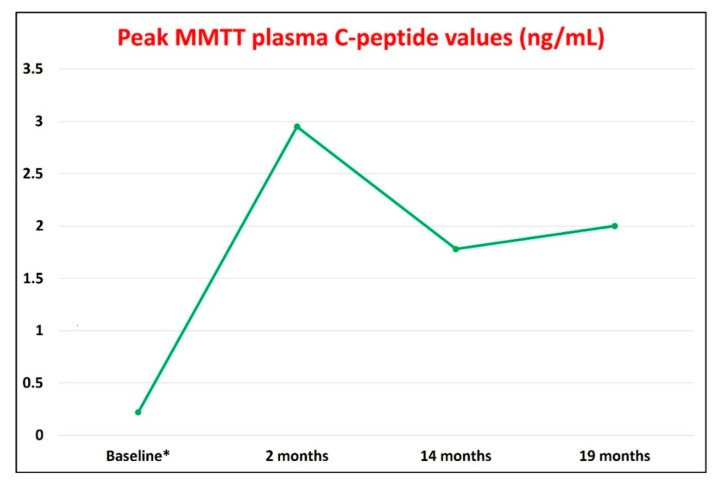
Peak plasma C-peptide values (ng/mL) observed during the 120 min mixed meal tolerance tests (MMTTs) performed at different timepoints of the follow-up period. Baseline timepoint corresponds to the occurrence of diabetic ketoacidosis and diagnosis of type 1 diabetes. * Only baseline C-peptide values refer to the fasting state.

We decided to perform another MMTT and other laboratory tests 3 months after the administration of the second COVID-19 vaccine dose (corresponding to 19 months after T1D onset). Fortunately, and unexpectedly, the patient remained in the honeymoon phase during this entire period. In fact, although there was a slight increase in HbA1c values compared to 5 months before (5.7% vs. 5.2%) as a likely consequence of the transient impairment of glucose control induced by the COVID-19 vaccine, we did not observe substantial changes in residual endogenous insulin secretion: peak plasma C-peptide values of 2.00 ng/mL, reached at 120 min; +0.22 ng/mL with respect to the peak plasma C-peptide values observed during the MMTT performed 5 months before (Figure 5; Supplementary Table S1). Moreover, we did not observe substantial changes in total daily insulin requirements (insulin degludec, 7 IU/day at bedtime, amounting to a total daily insulin dose of 0.10 IU/Kg body weight/24 h). The IDAA1c value of 6.1 confirmed the persistence of the honeymoon phase (Supplementary Table S1). The GADA titer remained stable over time, with comparable levels observed at baseline and 19 months after the T1D diagnosis (24.6 AU/mL vs. 26.3 AU/mL, respectively; Supplementary Table S1). In addition, during the entire follow-up period, serum 25-hydroxyvitamin D levels remained >40 ng/mL (Supplementary Table S1), which is considered the target circulating value for the achievement of the anti-inflammatory and immunomodulatory actions and other extraskeletal benefits of vitamin D [24,36,37]. CBC and other laboratory test results were unremarkable (Supplementary Table S2). 

A verification panel for the determination of anti-SARS-CoV-2 antibodies based on chemiluminescent microparticle immunoassay (CMIA) was performed 3 months after the second vaccine dose administration and revealed high anti-SARS-CoV-2-neutralizing IgG antibody titers (anti-S1/RBD: 6909.37 BAU/mL; cut-off for positivity: ≥7.1), as well as the absence of anti-SARS-CoV-2 neutralizing IgM antibodies (anti-Spike Protein antibodies index: 0.26; cut-off for positivity: index ≥ 1.00). This finding indicated a strong humoral immune response to the two-dose regimen of the BNT162b2 mRNA vaccine. In this regard, it is worth noting that humoral immune response to COVID-19 vaccination is comparable in healthy controls and in patients with type 1 and type 2 diabetes, irrespective of glucose control [38].

## 3. Discussion

In this case report, we showed that the BNT162b2 mRNA COVID-19 vaccine does not negatively affect the honeymoon phase and the residual beta-cell function in T1D. Conversely, the transient alteration in blood glucose control observed after COVID-19 vaccination has already been reported in the literature. Although we presented data of a single case, the main strengths of our study are represented by the measurement of stimulated C-peptide levels over time and by the assessment of blood glucose patterns through continuous glucose monitoring (CGM) before and after COVID-19 vaccination. Moreover, this case is peculiar due to the prolonged honeymoon phase (considering an average duration of this phase of about 7 months) [16].

Studies have documented that some patients with T1D experience a transient instability of blood glucose levels post-vaccine administration, which usually fades within a few days [9,10]. These findings have initially suggested the existence of a possible link between immune and metabolic responses after COVID-19 vaccination in patients with T1D. More recently, an Italian cross-sectional study conducted on 30 T1D patients wearing a flash glucose monitoring (FGM) device showed that the third Pfizer-BioNTech COVID-19 vaccine dose (booster dose) significantly increased the glycemic variability (expressed as coefficient of variation) and the daily insulin requirements during the 7 days following the booster dose administration compared to the previous week [39]. Nevertheless, mean glucose values, TIR, TAR, and time below range (TBR) related to the 7 days after the booster dose administration did not significantly differ from those related to the previous week [39].

The possible mechanisms by which COVID-19 vaccines may cause hyperglycemia remain to be determined. Samuel et al. [7] hypothesized that ViHG may be caused by pancreatic injury and/or acute pancreatitis secondary to reduced pancreatic blood flow and increased cellular oxidative stress. According to this hypothesis, case reports of pancreatic injury/acute pancreatitis following the administration of the Pfizer-BioNTech COVID-19 mRNA vaccine have been described [40,41,42]. It is also plausible that the transient alteration in blood glucose levels is caused by vaccine-induced inflammation and immune response. Vaccines, by their own nature, elicit an immune response whose magnitude varies within and between individuals due to a series of factors, including the type of vaccine adjuvant or the host immune response genes. The transient alteration in blood glucose levels may be more pronounced in individuals with existing impaired glucose control. Of note, the rise in circulating levels of pro-inflammatory cytokines (e.g., TNF-α, IL-1, IL-6, IFN-γ) in response to different triggers (e.g., vaccine excipients, adenoviral vectors, or the vaccine-derived SARS-CoV-2 spike protein immunogen) may cause pancreatic beta-cell dysfunction/injury [43,44] and/or insulin resistance [45,46,47], resulting in an acute impairment of blood glucose levels [7,48,49].

Another (non-mutually exclusive) explanation of ViHG relies on the fact that the stimulation of the immune system following COVID-19 vaccination may cause a stress response associated with a rise in circulating levels of counterregulatory hormones such as growth hormone, adrenaline, cortisol, and/or glucagon [50,51]. Accordingly, there are reports of DKA development after administration of different COVID-19 vaccines, such as ChAdOx1 nCoV-19 vaccine and BBV152-whole virion inactivated vaccine [52], mRNA-1273 Moderna vaccine [53], and BNT162b2 (Pfizer-BioNTech) mRNA vaccine [54], in the absence of other precipitating factors for DKA. The activation of systemic inflammatory pathways and the rise in circulating levels of counterregulatory hormones after COVID-19 vaccine administration may substantially contribute to hyperglycemic emergencies such as DKA and/or HHS as a likely consequence of increased insulin resistance and hepatic glucose production [45,46,47,55]. Yet, in our case, fasting capillary blood ketones remained negative at the follow-up visits performed after COVID-19 vaccination. Moreover, the patient did not experience polyuria, nocturia, or polydipsia after the vaccination. It is also worth specifying that almost all CGM metrics remained within the optimal target values established for T1D patients according to the International Consensus on Time in Range [56]. Only TAR > 250 mg/dL was above the optimal target values (7%; target value: <5%); however, this alteration persisted only during the 21 days after the second vaccine dose injection. Then, TAR > 250 mg/dL returned within the target values (2%) (Supplementary Table S3, Figure 3 and Figure 4).

Recently, there has also been increasing concern that COVID-19 vaccines may trigger the onset of autoimmune diseases through molecular mimicry, the production of autoantibodies, and/or via the action of certain vaccine adjuvants [57]. There are case reports of autoimmune endocrine diseases that have occurred after COVID-19 vaccination, such as Graves’ disease [58,59] and T1D (discussed later in the text). It has been hypothesized that these disorders occur as a result of different mechanisms, including autoimmune/autoinflammatory syndrome induced by adjuvants (ASIA), mRNA “self-adjuvant” properties, immune disruption from external stimuli, and molecular mimicry between viral antigen proteins and human proteins (e.g., beta-cell antigens) [58]. In this regard, there have been case reports of adult-onset autoantibody-negative fulminant T1D [60,61] and autoantibody-positive T1D [62,63] that occurred after the administration of different COVID-19 vaccine types and were characterized by severe insulinopenia and/or DKA development. Sasaki et al. [64] hypothesized that two different pathophysiological mechanisms may underlie these two distinct forms of T1D observed after COVID-19 vaccination. Notably, the fulminant occurrence of T1D (3–7 days after vaccination) associated with the negativity of islet autoantibodies may suggest direct damage to beta cells caused by SARS-CoV-2 spike proteins and/or by the vaccine-induced rise in pro-inflammatory cytokines [64]. Indeed, it has been suggested that direct SARS-CoV-2 infection can trigger apoptosis and the transdifferentiation of beta cells [65]. Conversely, a longer time window between vaccine administration and T1D onset (4–7 weeks after the first or second vaccine dose administration), associated with the positivity of islet autoantibodies, suggests an autoimmune etiology, which requires a longer period for the process of cross-immunity [63,64]. Therefore, COVID-19 vaccination of nondiabetic individuals who have a genetic susceptibility to T1D may theoretically increase the risk of developing clinical T1D [64], as vaccination may evoke islet autoimmunity and cause irreversible pancreatic beta-cell destruction in such individuals. However, it is important to take into account that SARS-CoV-2 may cause beta-cell damage through direct beta-cell virus tropism and/or putative infection of pancreatic microvasculature and/or ductal cells [66]. Moreover, the increased prevalence of insulin-requiring hyperglycemia in patients with COVID-19 suggests that SARS-CoV-2 may trigger selective beta-cell inflammation [67], which could predispose to beta-cell autoimmunity and contribute to the complex pathogenesis of T1D [68]. On the other hand, in patients with established T1D, the low beta-cell secretory reserve may confer a high risk for DKA development under SARS-CoV-2 infection, potentially leading to higher disease morbidity and mortality [69].

Although our patient was already diagnosed with T1D, the derangements in blood glucose levels started approximately 10 days after the first vaccine dose injection and became more pronounced after the second vaccine dose administration, resulting in the need to resume prandial insulin injections for one week. The more pronounced exacerbation of hyperglycemia observed after the second vaccine dose administration may suggest that transient ViHG was mainly immune-mediated. Indeed, the second COVID-19 vaccine dose, as compared to the first one, can trigger a strong and more robust immediate innate immune response followed by an adaptive immune response [70]. Accordingly, our patient showed high anti-SARS-CoV-2-neutralizing IgG antibody titers 3 months after the second vaccine dose administration, indicating a strong humoral immune response to the COVID-19 vaccine. Nonetheless, the GADA titer measured at the same time point (3 months after the second vaccine dose injection) remained stable (Supplementary Table S1). The latter findings seem to exclude an autoimmune etiology of the ViHG in our patient. In addition, beta-cell dysfunction caused by the vaccine (through the damage to beta cells mediated by increased circulating levels of pro-inflammatory cytokines, vaccine excipients, and/or vaccine-derived SARS-CoV-2 spike protein immunogen) may also be excluded in our case due to the fact that peak MMTT plasma C-peptide levels did not decrease but rather increased 3 months after the second vaccine dose administration (Supplementary Table S1, Figure 5). Notably, the observed peak MMTT plasma C-peptide level (2.00 ng/mL) characterizes the high stimulated C-peptide subgroup of T1D patients, which is defined by a peak MMTT C-peptide level >1.20 ng/mL according to the classification proposed by Dr. Rickels and colleagues [71]. 

In view of the above, it may be speculated that, in our case, the transient 6-week ViHG could have been caused by a temporary (vaccine-induced) increase in circulating levels of pro-inflammatory cytokines, with a subsequent increase in peripheral insulin resistance leading to short-term metabolic derangements. This hypothesis may also shed light on the pathophysiology of the honeymoon phase of T1D. Indeed, C-peptide levels and GADA titers were not substantially affected by the vaccination, and blood glucose values returned to near-normal values approximately 3 weeks after the second vaccine dose administration. These findings might suggest that the maintenance of the honeymoon phase in T1D is primarily driven by immune factors (transient recovery of immune tolerance to beta-cell autoantigens) rather than by metabolic factors. Yet, mechanistic studies are needed to confirm this hypothesis.

## 4. Conclusions

In conclusion, we first showed that BNT162b2 mRNA COVID-19 vaccine caused only a transient ViHG without altering the course of the honeymoon phase in a young adult patient with T1D. Indeed, BNT162b2 mRNA COVID-19 vaccine did not affect the residual beta-cell function, nor did it evoke or exacerbate islet autoimmunity. This finding may prompt clinicians and diabetologists to encourage COVID-19 vaccination even in T1D patients during the honeymoon phase, thus overcoming the vaccine hesitancy observed in many of these subjects [72,73]. However, it is important to outline that large prospective studies are needed to confirm the safety and the immunometabolic impact of the BNT162b2 mRNA COVID-19 vaccine in T1D patients during the honeymoon phase.

We believe that T1D patients who are going to receive COVID-19 vaccination should be counseled and warned about the possible occurrence of transient ViHG. These patients should undergo strict postvaccination surveillance through outpatient visits and/or telemedicine with remote glucose monitoring by clinicians and diabetologists. Yet, the possible occurrence of ViHG should in no way contribute to vaccine hesitancy, nor should it be a reason to withhold COVID-19 vaccination.

## Data Availability

All data analyzed in this study are included in the present article and its Supplementary Material.

## Supplementary Materials

The following supporting information can be downloaded at: https://www.mdpi.com/article/10.3390/vaccines10071096/s1, Table S1: Anthropometric parameters, blood pressure values and markers of glucose homeostasis, residual beta-cell function and beta-cell autoimmunity during the follow-up period; Table S2: Laboratory tests (other than markers of glucose homeostasis, residual beta-cell function and beta-cell autoimmunity) performed at 2 months, 14 months and 19 months from the clinical onset of type 1 diabetes (T1D); Table S3: Summary of the main continuous glucose monitoring (CGM) metrics during different periods before and after COVID-19 vaccination.
