# Supplementary material for: BNT162b2 mRNA COVID-19 Vaccine Does Not Impact the Honeymoon Phase in Type 1 Diabetes: A Case Report
Source: Vaccines (Basel). 2022 Jul 8;10(7):1096. doi: 10.3390/vaccines10071096 (PMC9319173; doi:10.3390/vaccines10071096)
Supplement: Supplementary file 1 [file vaccines-10-01096-s001.zip › vaccines-1770666-supplementary.pdf]

vaccines

MDPI

**Supplementary Materials**

**Table S1.** Anthropometric parameters, blood pressure values and markers of glucose homeostasis, residual beta-cell function and beta-cell autoimmunity during the follow-up period.

| Parameter | T1D onset | 2 months after the T1D onset | 14 months after the T1D onset | 19 months after the T1D onset (3 months after the second COVID-19 vaccine dose) |
|---|---|---|---|---|
| Body weight (Kg) | 69 | 71.5 | 67.5 | 68.5 |
| BMI (Kg/m$^2$) | 22.5 | 23.3 | 21.8 | 22.1 |
| Blood pressure (mmHg) | 130/90 | 120/60 | 110/70 | 120/70 |
| HbA1c (%; mmol/mol) | 13.2% (121 mmol/mol) | 5.3% (34 mmol/mol) | 5.2% (33 mmol/mol) | 5.7% (39 mmol/mol) |
| Total daily insulin dose (TDI) | 0.7 IU/ body weight/24 hours (after DKA resolution; basal-bolus insulin therapy) | 0.09 IU/Kg body weight/24 hours (insulin degludec 7 IU/day) | 0.10 IU/Kg body weight/24 hours (insulin degludec 7 IU/day) | 0.10 IU/Kg body weight/24 hours (insulin degludec 7 IU/day) |
| IDAA1c | Not applicable | 5.6 | 5.6 | 6.1 |
| Fasting capillary blood ketones (mmol/L) | >3.0 | 0.3 | 0.2 | 0.3 |
| Fasting plasma C-peptide (ng/mL) (reference range: 0.78–5.19) | 0.22 | 0.74 | 0.87 | 0.28 |
| 30-min (MMTT) plasma C-peptide (ng/mL) | n/a | 1.89 | 1.52 | 0.90 |
| 60-min (MMTT) plasma C-peptide (ng/mL) | n/a | 2.53 | 1.62 | 1.25 |
| 90-min (MMTT) plasma C-peptide (ng/mL) | n/a | 2.95 | 1.69 | 1.52 |
| 120-min (MMTT) plasma C-peptide (ng/mL) | n/a | 2.53 | 1.78 | 2.00 |
| Fasting plasma glucose (mg/dL) | 501 | 80 | 72 | 72 |
| 30-min (MMTT) plasma glucose (mg/dL) | n/a | 203 | 146 | 170 |
| 60-min (MMTT) plasma glucose (mg/dL) | n/a | 151 | 183 | 235 |
| 90-min (MMTT) plasma glucose (mg/dL) | n/a | 110 | 165 | 261 |
| 120-min (MMTT) plasma glucose (mg/dL) | n/a | 81 | 182 | 250 |

| | | | | |
|---|---|---|---|---|
| Fasting C-peptide index | 0.04 | 0.92 | 1.20 | 0.38 |
| 30-min (MMTT) C-peptide index | n/a | 0.93 | 1.04 | 0.52 |
| 60-min (MMTT) C-peptide index | n/a | 1.67 | 0.88 | 0.53 |
| 90-min (MMTT) C-peptide index | n/a | 2.68 | 1.02 | 0.58 |
| 120-min (MMTT) C-peptide index | n/a | 3.12 | 0.97 | 0.80 |
| GADA (reference range: 0.00–5.00 AU/mL) | 24.6 | n/a | 26.0 | 26.3 |
| IAA (reference range: 0.00–9.00 U/mL) | 5.1 | n/a | 1.8 | 0.4 |
| IA2A (cut-off for positivity: ≥7.4 U/mL) | >350.0 | n/a | 201.10 | n/a |
| ZnT8A (cut-off for positivity: ≥14.9 U/mL) | 18.9 | n/a | 9.60 | n/a |
| ICA (cut-off for negativity - indirect immunofluorescence: <1:5) | <1:5 | n/a | <1:5 | <1:5 |
| Serum 25-hydroxyvitamin D levels (ng/mL) | n/a | 28.0 | 57 | 45 |

IDAA1c (Insulin-Dose Adjusted HbA1c) was calculated through the following formula: HbA1c (%) + 4 x [insulin dose (units per kilogram per 24 h)]. A calculated IDAA1C value of ≤9 was used to define the partial remission [ref. 21]. 120-minute-mixed meal tolerance tests (MMTTs) were performed via oral administration of Ensure Plus®, Abbott (360 mL) for the assessment of residual beta-cell function. C-peptide index (also referred to as C-peptide to glucose ratio) was assessed in fasting and (MMTT)-postprandial states, and was calculated through the following formula: C-peptide (ng/mL)/glucose (mg/dL) (×100) [ref. 23]. **Abbreviations**: BMI, body mass index; GADA, glutamic acid decarboxylase autoantibodies; HbA1c, glycated hemoglobin; IAA, insulin autoantibodies; IA2A, islet tyrosine phosphatase 2 autoantibodies; ICA, islet-cell antibodies; IDAA1c, Insulin-Dose Adjusted HbA1c; MMTT, mixed meal tolerance test; n/a, not available; T1D, type 1 diabetes; ZnT8A, zinc transporter 8 autoantibodies.

**Table S2.** Laboratory tests (other than markers of glucose homeostasis, residual beta-cell function and beta-cell autoimmunity) performed at 2 months, 14 months and 19 months from the clinical onset of type 1 diabetes (T1D).

| Laboratory parameter * | 2 months after the T1D onset | 14 months after the T1D onset | 19 months after the T1D onset ** |
|---|---|---|---|
| WBC count (4.00–10.00×10³/μL) | 5.34 × 10³/μL | 5.71×10³/μL | 6.23×10³/μL |
| Neutrophils (1.8–7.0×10³/μL; 40.0–75.0%) | 2.80×10³/μL (52.2%) | 3.01×10³/μL (52.8%) | 3.12×10³/μL (50.0%) |

| | | | |
|---|---|---|---|
| Lymphocytes (1.0–4.8×10³/μL; 19.0–48.0%) | 2.10×10³/μL (40.0%) | 2.19×10³/μL (38.40%) | 2.59×10³/μL (41.50%) |
| Monocytes (0.1–0.8×10³/μL; <12.0%) | 0.3×10³/μL (5.0%) | 0.36×10³/μL (6.3%) | 0.38×10³/μL (6.2%) |
| Eosinophils (0.0–0.5×10³/μL; <6.0%) | 0.1×10³/μL (1.7%) | 0.12×10³/μL (2.1%) | 0.08×10³/μL (1.3%) |
| Basophils (0.0–0.2×10³/μL; <2.0%) | 0.1×10³/μL (1.0%) | 0.03×10³/μL (0.5%) | 0.07×10³/μL (1.1%) |
| RBC count (4.40–5.90×10⁶/μL) | 5.21×10⁶/μL | 5.07×10⁶/μL | 5.11×10⁶/μL |
| Hb (14.0–18.0 g/dL) | 15.7 g/dL | 15.1 g/dL | 15.3 g/dL |
| Ht (41–51%) | 46% | 43.2% | 44.5% |
| MCV (82.0–98.0 fl) | 88.9 fl | 85.2 fl | 87.0 fl |
| MCH (27.0–34.0 pg) | 30.1 pg | 29.7 pg | 29.9 pg |
| MCHC (32.0–36.0 g/dL) | 33.9 g/dL | 34.9 g/dL | 34.3 g/dL |
| Platelet count (150–450×10³/μL) | 153x10³/μL | 160x10³/μL | 163x10³/μL |
| BUN (19–44 mg/dL) | 35 mg/dL | 38 mg/dL | 39 mg/dL |
| Creatinine (0.73–1.18 mg/dL) | 1.05 mg/dL | 0.84 mg/dL | 1.13 mg/dL |
| eGFR (CKD-EPI) | 100.3 mL/min/1.73m² | 122.6 mL/min/1.73m² | 90.5 mL/min/1.73m² |
| Uric acid (3.57–7.20 mg/dL) | 5.5 mg/dL | 5.0 mg/dL | 5.0 mg/dL |
| Total serum protein level (60–80 g/L) | 65 g/L | 63 g/L | 68 g/L |
| Total bilirubin (<1.20 mg/dL) | 0.63 mg/dL | 0.54 mg/dL | 1.15 mg/dL |
| Direct bilirubin (<0.5 mg/dL) | 0.36 mg/dL | 0.25 mg/dL | 0.40 mg/dL |
| AST (5–34 U/L) | 19 U/L | 22 U/L | 23 U/L |
| ALT (0–55 U/L) | 13 U/L | 11 U/L | 14 U/L |
| GGT (12–64 U/L) | 12 U/L | 12 U/L | 13 U/L |
| ALP | | | 48 U/L |

| | | | |
|---|---|---|---|
| (40–150 U/L) | 58 U/L | 62 U/L | |
| Sodium (135–145 mmol/L) | 142 mmol/L | 141 mmol/L | 139 mmol/L |
| Potassium (3.5–5.1 mmol/L) | 3.63 mmol/L | 3.8 mmol/L | 3.7 mmol/L |
| Calcium (8.4–10.2 mg/dL) | 8.9 mg/dL | 8.8 mg/dL | 9.4 mg/dL |
| Phosphorus (2.3–4.7 mg/dL) | 3.3 mg/dL | 3.3 mg/dL | 4.0 mg/dL |
| Magnesium (1.6–2.6 mg/dL) | 2.2 mg/dL | 2.3 mg/dL | 2.2 mg/dL |
| Serum iron (65–175 μg/dL) | 70 μg/dL | 68 μg/dL | 155 μg/dL |
| Transferrin (174–364 mg/dL) | 211 mg/dL | 219 mg/dL | 213 mg/dL |
| Ferritin (21.8–275 ng/mL) | 132 ng/mL | 100 ng/mL | 107 ng/mL |
| Albumin (35–50 g/L) | 42 g/L | 42.8 g/L | 46.6 g/L |
| Total cholesterol (<200 mg/dL) | 146 mg/dL | 132 mg/dL | 160 mg/dL |
| HDL-cholesterol (>40 mg/dL) | 42 mg/dL | 49 mg/dL | 54 mg/dL |
| LDL-cholesterol (<130 mg/dL) | 95 mg/dL | 77 mg/dL | 102 mg/dL |
| Triglycerides (<150 mg/dL) | 46 mg/dL | 38 mg/dL | 26 mg/dL |
| TSH (0.35–4.94 μIU/mL) | 1.07 μIU/mL | 1.41 μIU/mL | 1.26 μIU/mL |
| FT3 (1.72–3.54 pg/mL) | 2.74 pg/mL | 2.46 pg/mL | 2.83 pg/mL |
| FT4 (0.7–1.48 ng/dL) | 0.90 ng/dL | 0.88 ng/dL | 1.04 ng/dL |
| Anti-thyroid peroxidase (TPO) antibodies (0.0–5.6 IU/mL) | 0.0 IU/mL | n/a | 0.2 IU/mL |
| Antithyroglobulin antibodies (0.0–4.1 IU/mL) | 2.0 IU/mL | n/a | 0.9 IU/mL |
| Tissue transglutaminase IgA antibodies (<10 AU/mL) | 2.40 AU/mL | 2.30 AU/mL | 2.50 AU/mL |
| Total IgA (0.70–3.78 g/L) | n/a | 1.15 g/L | 1.10 g/L |
| Total testosterone (2.40–8.70 ng/mL) | 4.02 ng/mL | 4.38 ng/mL | 3.37 ng/mL |
| Serum cortisol - 08:00 AM | n/a | 13.3 μg/dL | n/a |

| | | | |
|---|---|---|---|
| (3.7–19.4 µg/dL) | | | |
| ACTH - 08:00 AM (4.7–48.8 pg/mL) | n/a | 15 pg/mL | n/a |
| Folate (3.1–20.5 ng/mL) | 5.5 ng/mL | 4.8 ng/mL | 9.2 ng/mL |
| Vitamin B12 (187–883 pg/mL) | 442 pg/mL | 389 pg/mL | 509 pg/mL |
| 25(OH)D (≥30 ng/mL) | 28 ng/mL | 57 ng/mL | 45 ng/mL |
| PTH (15.0–83.1 pg/mL) | n/a | 40.6 pg/mL | 48.6 pg/mL |
| Urinalysis | Absence of glucose, proteins, ketones and nitrites in urine; Albumin-to-creatinine ratio (ACR) 15 mg/g | Absence of glucose, proteins, ketones and nitrites in urine; Albumin-to-creatinine ratio (ACR) 15 mg/g | Absence of glucose, proteins, ketones and nitrites in urine; Albumin-to-creatinine ratio (ACR) 15 mg/g |

* Reference ranges at our Institution are shown in brackets. ** 3 months after the second COVID-19 vaccine dose injection. **Abbreviations**: 25(OH)D, 25-hydroxyvitamin D; ACTH, adrenocorticotropic hormone; ALP, alkaline phosphatase; ALT, alanine aminotransferase; AST, aspartate aminotransferase; BUN, blood urea nitrogen; eGFR (CKD-EPI), estimated glomerular filtration rate calculated through the CKD-EPI (Chronic Kidney Disease Epidemiology Collaboration) equation; FT3, free triiodothyronine; FT4, free thyroxine; GGT, gamma-glutamyl transferase; Hb, hemoglobin; HDL-cholesterol, high-density lipoprotein cholesterol; Ht, hematocrit; IgA, Immunoglobulin A; LDL-cholesterol, low-density lipoprotein cholesterol; MCH, mean corpuscular hemoglobin; MCHC, mean corpuscular hemoglobin concentration; MCV, mean corpuscular volume; PTH, parathyroid hormone; RBC, red blood cell count; TSH, thyroid-stimulating hormone; WBC, white blood cell count.

**Table S3.** Summary of the main continuous glucose monitoring (CGM) metrics during different periods before and after COVID-19 vaccination.

| **CGM metrics** | **23 Nov 2021 - 13 Dec 2021** (21 days before the first vaccine dose) | **14 Dec 2021 - 3 Jan 2022** (21 days after the first vaccine dose) | **4 Jan 2022 - 24 Jan 2022** (21 days after the second vaccine dose) | **25 Jan 2022 - 14 Feb 2022** (21 days following the first 3 weeks after the second vaccine dose) |
|---|---|---|---|---|
| TIR 70–180 mg/dL | 90% | 87% | 74% | 88% |
| TAR 181–250 mg/dL | 8% | 11% | 19% | 9% |
| TAR>250 mg/dL | 2% | 2% | 7% | 2% |
| TBR 54–69 mg/dL | 0% | 0% | 0% | 1% |
| TBR<54 mg/dL | 0% | 0% | 0% | 0% |

| | | | | |
|---|---|---|---|---|
| GMI | 6.4% (47 mmol/mol) | 6.6% (49 mmol/mol) | 7.0% (53 mmol/mol) | 6.3% (46 mmol/mol) |
| Glycemic variability (%CV) | 29.9% | 28.8% | 32.8% | 33.0% |
| Percentage of time CGM was active | 91% | 92% | 83% | 79% |

**Abbreviations**: CV, coefficient of variation; GMI, Glucose Management Indicator (also referred to as estimated A1C or eA1C); TAR, time above range; TBR, time below range; TIR, time in range.
